# The genome sequence of the Northern Deep-brown Dart, *Aporophyla lueneburgensis* (Freyer, 1848)

**DOI:** 10.12688/wellcomeopenres.19296.1

**Published:** 2023-03-31

**Authors:** Douglas Boyes, Peter W.H. Holland, University of Oxford and Wytham Woods Genome Acquisition Lab, Darwin Tree of Life Barcoding collective, Wellcome Sanger Institute Tree of Life programme, Wellcome Sanger Institute Scientific Operations: DNA Pipelines collective, Tree of Life Core Informatics collective, Darwin Tree of Life Consortium

**Affiliations:** 1 UK Centre for Ecology & Hydrology, Wallingford, England, UK; 2 University of Oxford, Oxford, England, UK

**Keywords:** Aporophyla lueneburgensis, Northern Deep-brown Dart, genome sequence, chromosomal, Lepidoptera

## Abstract

We present a genome assembly from an individual female *Aporophyla lueneburgensis *(the Northern Deep-brown Dart; Arthropoda; Insecta; Lepidoptera; Noctuidae). The genome sequence is 978.3 megabases in span. Most of the assembly is scaffolded into 31 chromosomal pseudomolecules, including the Z sex chromosome. The mitochondrial genome has also been assembled and is 15.5 kilobases in length. Gene annotation of this assembly on Ensembl identified 12,580 protein coding genes.

## Species taxonomy

Eukaryota; Metazoa; Ecdysozoa; Arthropoda; Hexapoda; Insecta; Pterygota; Neoptera; Endopterygota; Lepidoptera; Glossata; Ditrysia; Noctuoidea; Noctuidae; Xyleninae; *Aporophyla*; *Aporophyla lueneburgensis* (Freyer, 1848) (NCBI:txid1337163).

## Background

*Aporophyla* is a genus of moths from the family Noctuidae found predominantly in Europe; most species in the genus have autumn flight periods. The Northern deep-brown dart *A. lueneburgensis* has brown or grey-brown forewings with a series of wavy markings forming a central darker band finely outlined in cream. The moth is widely distributed across Scotland and northern counties of England, with scattered and less frequent records from southern counties of England, Northern Ireland and Ireland ( NBN Atlas Partnership, 2021; Randle *et al.*, 2019; Thompson & Nelson, 2003). Although most common in northern latitudes of Europe and Scandinavia, the moth has also been recorded in Italy, Spain and Portugal ( Corley *et al.*, 2018; GBIF Secretariat, 2022). These reported distributions need further verification, as discussed below. *A. lueneburgensis* is univoltine with adults on the wing in August and September, often in moorland and rough grassland habitats. Larvae feed on heather *Calluna vulgaris* or bird’s-foot trefoil *Lotus corniculata* in autumn and again in spring, overwintering at an early larval stage ( Waring *et al.*, 2017).

There has been taxonomic debate about whether *A. lueneburgensis* should be given species status. For a century, the moth now named *A. lueneburgensis* was described as a colour variant of the deep-brown dart *Aporophyla lutulenta*, and was considered either a subspecies or given a variety designation, var. *luneburgensis*. In the 1950s, it was proposed that the two forms could be different species ( Wightman, 1954). “I am now quite satisfied that… two distinct species are involved” wrote Archibald Wightman, although confusingly he added “I can give no structural point of difference but I can say they are distinct” ( Wightman, 1954). The species-level separation was not initially adopted (for example, South, 1961), but gradually found favour through the second half of the 20th century (for example, Skinner & Wilson, 2009; Waring *et al.*, 2017). Distinctiveness of the two species was challenged from initial mitochondrial DNA barcode analyses ( Orhant, 2012), before being supported after DNA barcodes from more specimens were obtained ( Corley *et al.*, 2018; Haslberger & Segerer, 2016). Recent molecular analyses clearly support the view that *A. lueneburgensis* and *A. lutulenta* are indeed distinct species, although more specimens need to be analysed to determine accurately their geographic distribution ( Boyes *et al.*, 2021).

Here we report the complete genome sequence of *A. lueneburgensis*. In phylogenetic analyses, the mitochondrial CO1 DNA barcode of the specimen used here groups in a clade with other *A. lueneburgensis* specimens, distinct from *A. lutulenta* ( Boyes *et al.*, 2021). A complete genome sequence will facilitate studies into colour pattern evolution and adaptation to specific food plants, and contribute to research into lepidopteran genome evolution.

## Genome sequence report

**Figure 1.**
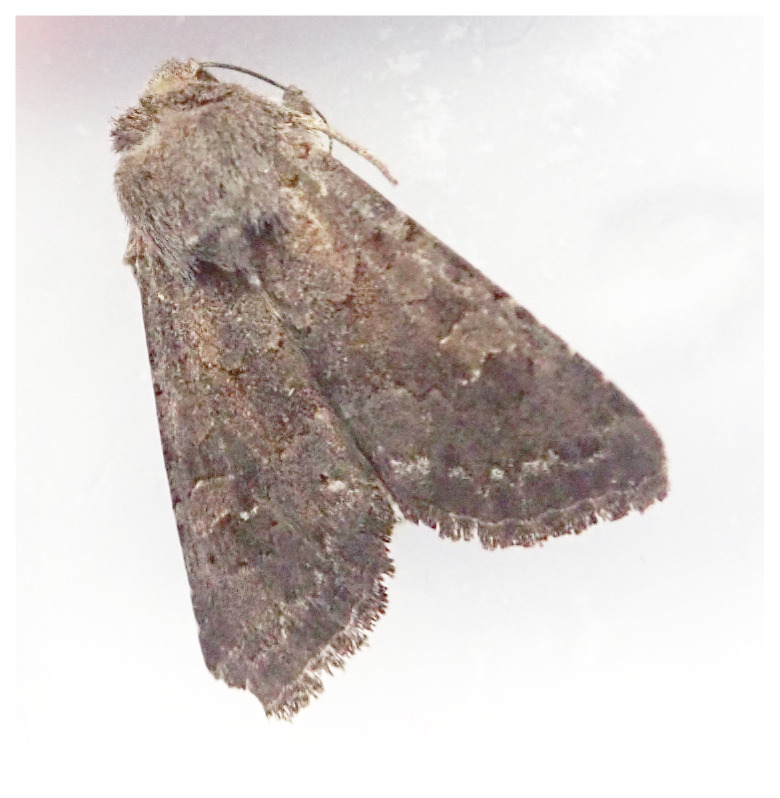
Photograph of the *Aporophyla lueneburgensis* (ilApoLuen1) specimen used for genome sequencing.

The genome was sequenced from one female *Aporophyla lueneburgensis* ( Figure 1) collected from Wytham Woods, Oxfordshire, UK (latitude 51.77, longitude –1.34). A total of 34-fold coverage in Pacific Biosciences single-molecule HiFi long reads was generated. Primary assembly contigs were scaffolded with chromosome conformation Hi-C data. Manual assembly curation corrected 33 missing or mis-joins, and removed 14 haplotypic duplications, reducing the assembly length by 1.27% and the scaffold number by 13.64%, and increasing the scaffold N50 by 0.63%.

**Figure 2.**
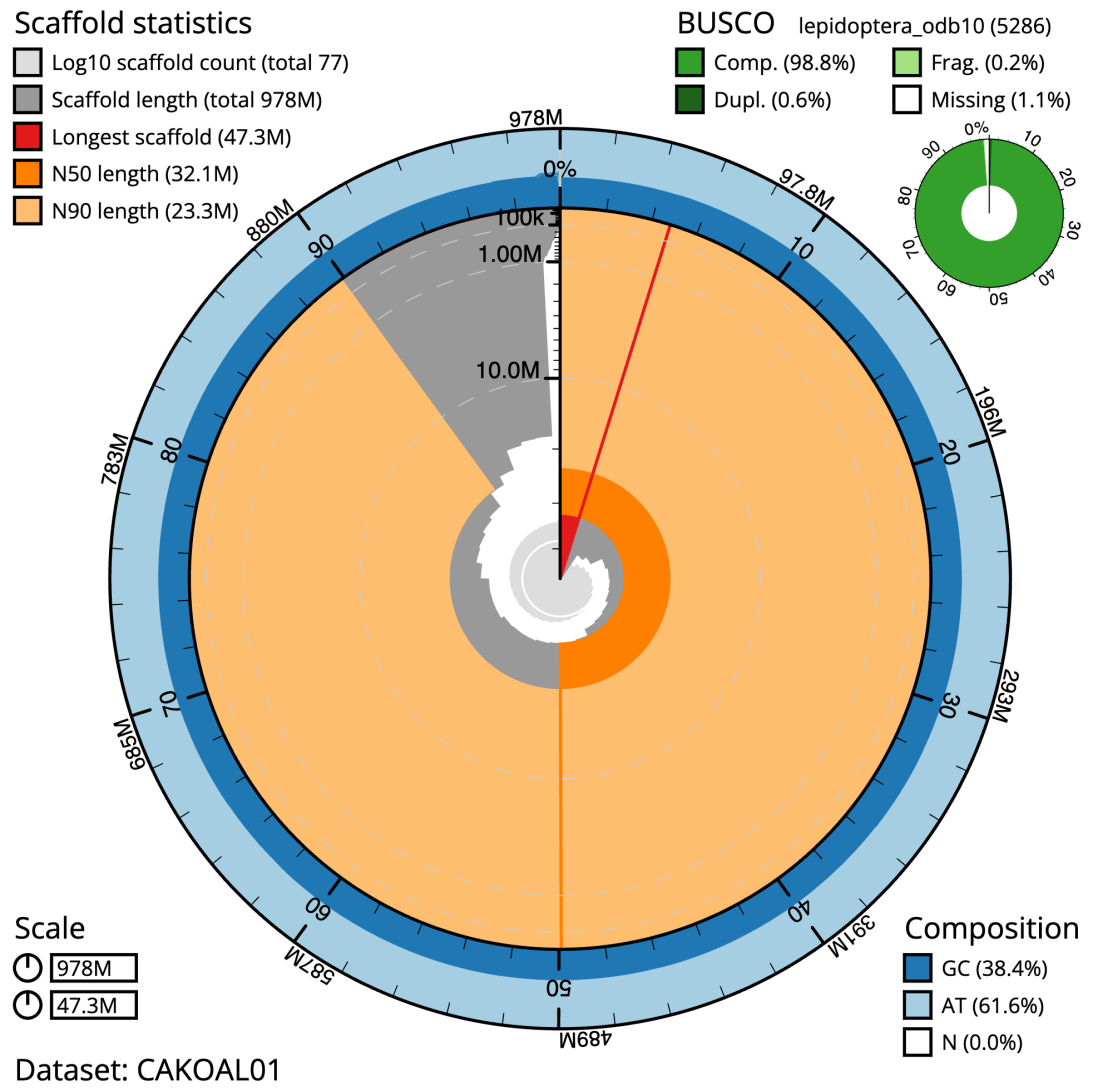
Genome assembly of *Aporophyla lueneburgensis*, ilApoLuen1.1: metrics. The BlobToolKit Snailplot shows N50 metrics and BUSCO gene completeness. The main plot is divided into 1,000 size-ordered bins around the circumference with each bin representing 0.1% of the 978,307,447 bp assembly. The distribution of scaffold lengths is shown in dark grey with the plot radius scaled to the longest scaffold present in the assembly (47,305,837 bp, shown in red). Orange and pale-orange arcs show the N50 and N90 scaffold lengths (32,091,479 and 23,324,438 bp), respectively. The pale grey spiral shows the cumulative scaffold count on a log scale with white scale lines showing successive orders of magnitude. The blue and pale-blue area around the outside of the plot shows the distribution of GC, AT and N percentages in the same bins as the inner plot. A summary of complete, fragmented, duplicated and missing BUSCO genes in the lepidoptera_odb10 set is shown in the top right. An interactive version of this figure is available at https://blobtoolkit.genomehubs.org/view/ilApoLuen1.1/dataset/CAKOAL01/snail.

The final assembly has a total length of 978.3 Mb in 76 sequence scaffolds with a scaffold N50 of 32.1 Mb ( Table 1). Most (99.12%) of the assembly sequence was assigned to 31 chromosomal-level scaffolds, representing 30 autosomes and the Z sex chromosome. Chromosome-scale scaffolds confirmed by the Hi-C data are named in order of size ( Figure 2– Figure 5; Table 2). There is half-coverage of the Z chromosome in the Hi-C map, but no W chromosome, indicating that the specimen is most likely a Z0 female ( Sahara *et al.*, 2012). While not fully phased, the assembly deposited is of one haplotype. Contigs corresponding to the second haplotype have also been deposited. The mitochondrial genome was also assembled and can be found as a contig within the multifasta file of the genome submission.

**Table 1.** Genome data for *Aporophyla lueneburgensis*, ilApoLuen1.1.

Project accession data		
Assembly identifier	ilApoLuen1.1	
Species	*Aporophyla lueneburgensis*	
Specimen	ilApoLuen1	
NCBI taxonomy ID	1337163	
BioProject	PRJEB50735	
BioSample ID	SAMEA8603194	
Isolate information	ilApoLuen1, thorax (genome sequencing), head (Hi-C scaffolding), abdomen (RNA sequencing)	
Assembly metrics *		*Benchmark*
Consensus quality (QV)	67.4	*≥ 50*
*k*-mer completeness	100%	*≥ 95%*
BUSCO **	C:98.8%[S:98.1%,D:0.6%], F:0.2%,M:1.1%,n:5,286	*C ≥ 95%*
Percentage of assembly mapped to chromosomes	99.12%	*≥ 95%*
Sex chromosomes	Z chromosome	*localised homologous pairs*
Organelles	Mitochondrial genome assembled	*complete single alleles*
Raw data accessions		
PacificBiosciences SEQUEL II	ERR8575368, ERR8575369	
Hi-C Illumina	ERR8571650	
PolyA RNA-Seq Illumina	ERR8571651	
Genome assembly		
Assembly accession	GCA_932294355.1	
*Accession of alternate haplotype*	GCA_932294405.1	
Span (Mb)	978.3	
Number of contigs	133	
Contig N50 length (Mb)	20.1	
Number of scaffolds	76	
Scaffold N50 length (Mb)	32.1	
Longest scaffold (Mb)	47.3	
**Genome annotation**		
Number of protein-coding genes	12,580	
Number of non protein-coding genes	1,675	
Number of gene transcripts	21,617	

* Assembly metric benchmarks are adapted from column VGP-2020 of “Table 1: Proposed standards and metrics for defining genome assembly quality” from ( Rhie *et al.*, 2021). ** BUSCO scores based on the lepidoptera_odb10 BUSCO set using v5.3.2. C = complete [S = single copy, D = duplicated], F = fragmented, M = missing, n = number of orthologues in comparison. A full set of BUSCO scores is available at https://blobtoolkit.genomehubs.org/view/ilApoLuen1.1/dataset/CAKOAL01/busco.

**Figure 3.**
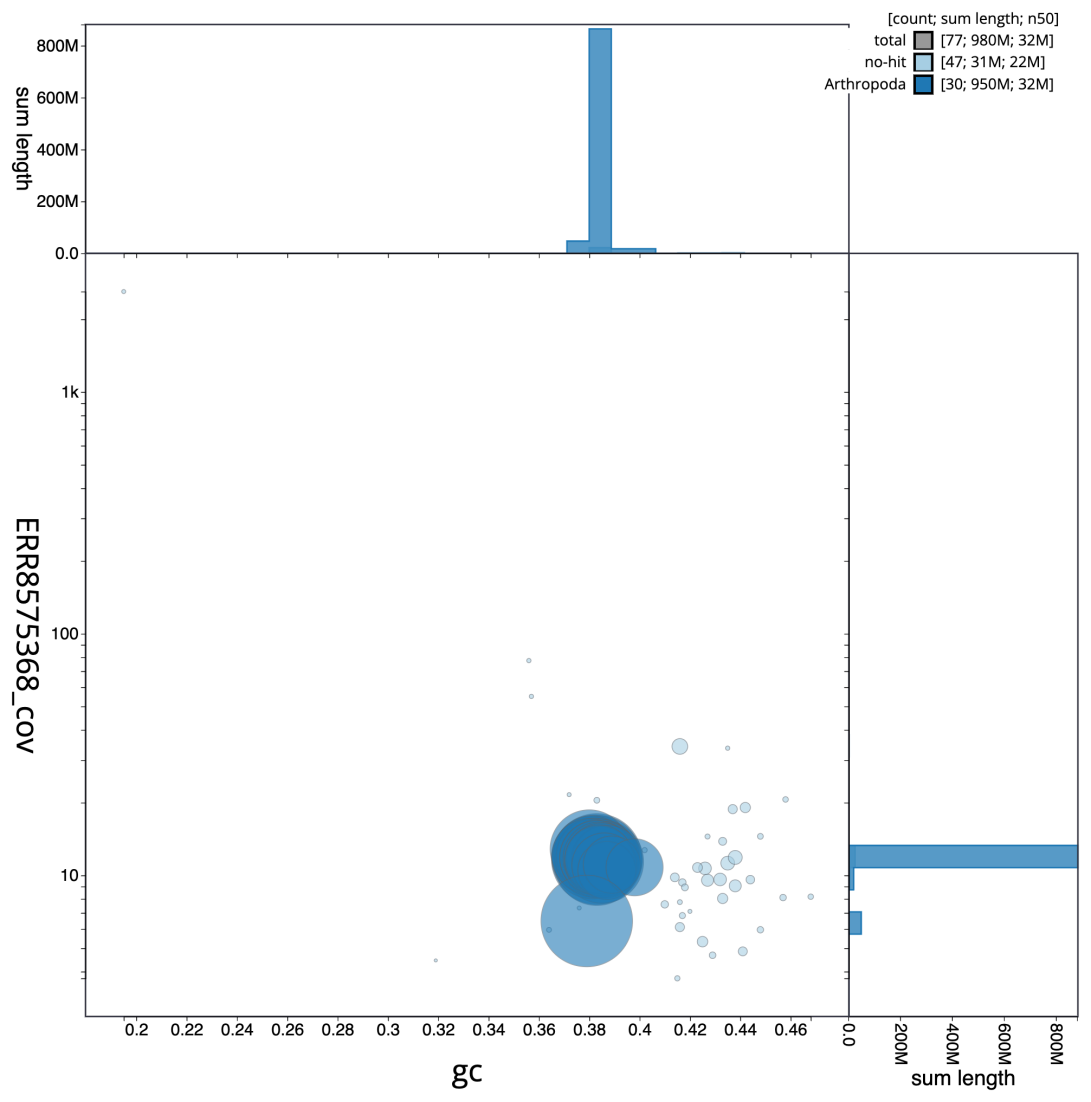
Genome assembly of *Aporophyla lueneburgensis*, ilApoLuen1.1: BlobToolKit GC-coverage plot. Scaffolds are coloured by phylum. Circles are sized in proportion to scaffold length. Histograms show the distribution of scaffold length sum along each axis. An interactive version of this figure is available at https://blobtoolkit.genomehubs.org/view/ilApoLuen1.1/dataset/CAKOAL01/blob.

**Figure 4.**
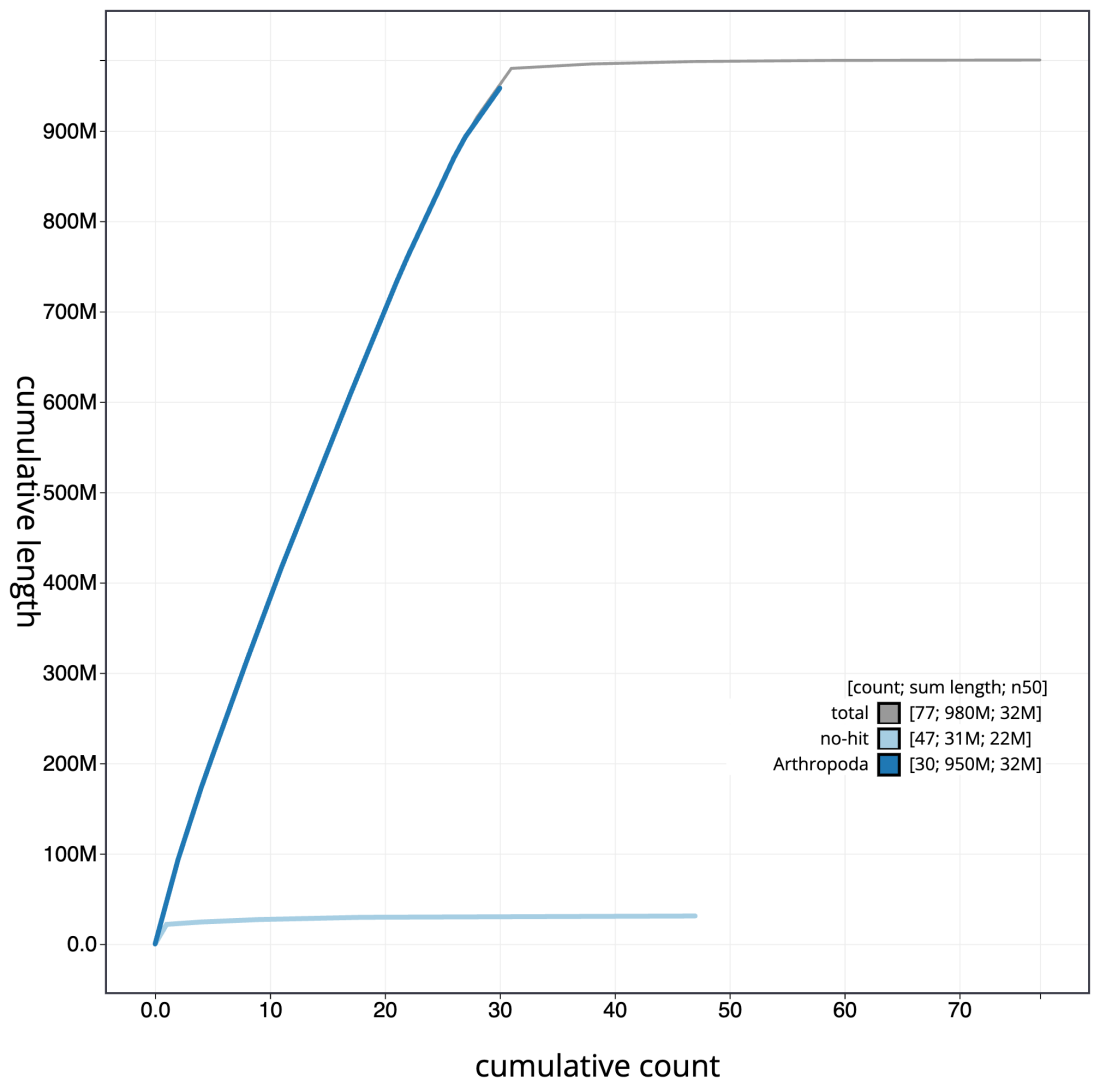
Genome assembly of *Aporophyla lueneburgensis*, ilApoLuen1.1: BlobToolKit cumulative sequence plot. The grey line shows cumulative length for all scaffolds. Coloured lines show cumulative lengths of scaffolds assigned to each phylum using the buscogenes taxrule. An interactive version of this figure is available at https://blobtoolkit.genomehubs.org/view/ilApoLuen1.1/dataset/CAKOAL01/cumulative.

**Figure 5.**
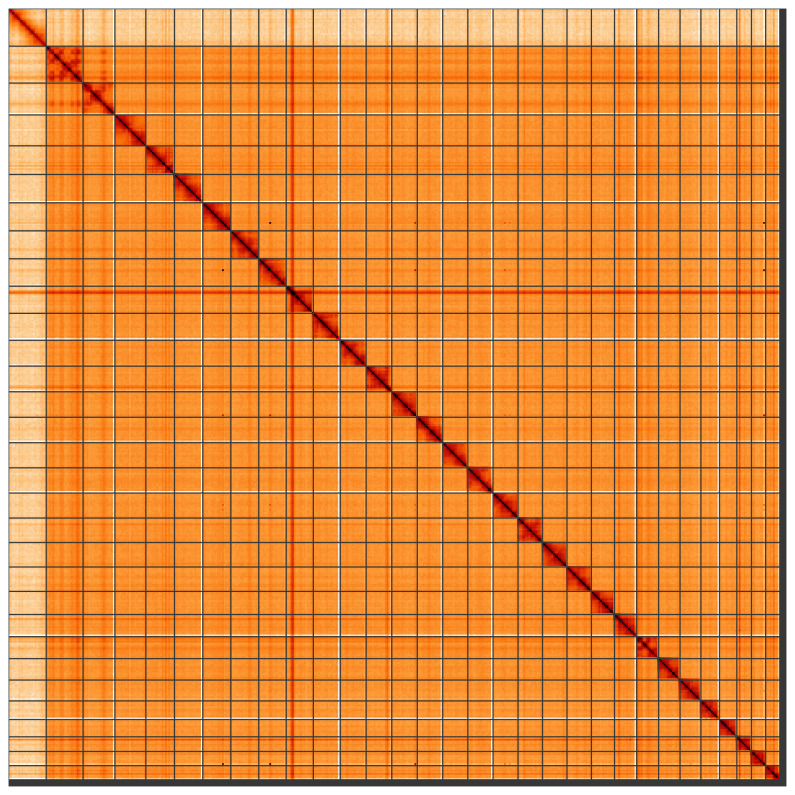
Genome assembly of *Aporophyla lueneburgensis*, ilApoLuen1.1: Hi-C contact map of the ilApoLuen1.1 assembly, visualised using HiGlass. Chromosomes are shown in order of size from left to right and top to bottom. An interactive version of this figure may be viewed at https://genome-note-higlass.tol.sanger.ac.uk/l/?d=W6Ke7WuBRfec0p-N2HNwbA.

**Table 2.** Chromosomal pseudomolecules in the genome assembly of *Aporophyla lueneburgensis*, ilApoLuen1.

INSDC accession	Chromosome	Size (Mb)	GC%
OW028734.1	1	46.33	38.3
OW028735.1	2	39.95	38.3
OW028736.1	3	38.89	38.5
OW028737.1	4	36.01	38.4
OW028738.1	5	35.5	38.1
OW028739.1	6	35.29	38.2
OW028740.1	7	35.11	38.1
OW028741.1	8	34.44	38.4
OW028742.1	9	34.14	38
OW028743.1	10	33.85	38.2
OW028744.1	11	32.41	38.4
OW028745.1	12	32.21	38.1
OW028746.1	13	32.09	38.2
OW028747.1	14	31.89	38.4
OW028748.1	15	31.64	38.4
OW028749.1	16	31.61	38.5
OW028750.1	17	31.47	38
OW028751.1	18	30.76	38.4
OW028752.1	19	30.74	38.3
OW028753.1	20	30.7	38.3
OW028754.1	21	29.04	38.4
OW028755.1	22	27.84	38.5
OW028756.1	23	27.54	38.7
OW028757.1	24	26.91	38.4
OW028758.1	25	26.24	38.4
OW028759.1	26	23.32	38.6
OW028760.1	27	21.75	38.7
OW028761.1	28	18.38	38.7
OW028762.1	29	17.88	38.9
OW028763.1	30	17.77	39.8
OW028733.1	Z	47.31	37.9
OW028764.1	MT	0.02	19.7
-	unplaced	9.27	42.8

The estimated Quality Value (QV) of the final assembly is 67.4 with *k*-mer completeness of 100%, and the assembly has a BUSCO v5.3.2 completeness of 98.8% (single = 98.1%, duplicated = 0.6%), using the lepidoptera_odb10 reference set ( *n* = 5,286).

Metadata for specimens, spectral estimates, sequencing runs, contaminants and pre-curation assembly statistics can be found here.

## Genome annotation report

The *Aporophyla lueneburgensis* genome assembly GCA_932294355.1 (ilApoLuen1.1) was annotated using the Ensembl rapid annotation pipeline ( Table 1; Accession number GCA_932294355.1). The resulting annotation includes 21,617 transcribed mRNAs from 12,580 protein-coding and 1,675 non-coding genes.

## Methods

### Sample acquisition and nucleic acid extraction

A female *Aporophyla lueneburgensis* (ilApoLuen1) was collected from Wytham Woods, Oxfordshire (biological vice-county: Berkshire), UK (latitude 51.77, longitude –1.34) on 8 September 2020. The specimen was taken from woodland habitat by Douglas Boyes (University of Oxford) using a light trap. The specimen was identified by the collector and snap-frozen on dry ice.

DNA was extracted at the Tree of Life laboratory, Wellcome Sanger Institute (WSI). The ilApoLuen1 sample was weighed and dissected on dry ice with head tissue set aside for Hi-C sequencing and abdomen tissue set aside for RNA sequencing. Thorax tissue was cryogenically disrupted to a fine powder using a Covaris cryoPREP Automated Dry Pulveriser, receiving multiple impacts. High molecular weight (HMW) DNA was extracted using the Qiagen MagAttract HMW DNA extraction kit. HMW DNA was sheared into an average fragment size of 12–20 kb in a Megaruptor 3 system with speed setting 30. Sheared DNA was purified by solid-phase reversible immobilisation using AMPure PB beads with a 1.8X ratio of beads to sample to remove the shorter fragments and concentrate the DNA sample. The concentration of the sheared and purified DNA was assessed using a Nanodrop spectrophotometer and Qubit Fluorometer and Qubit dsDNA High Sensitivity Assay kit. Fragment size distribution was evaluated by running the sample on the FemtoPulse system.

RNA was extracted from abdomen tissue of ilApoLuen1 in the Tree of Life Laboratory at the WSI using TRIzol, according to the manufacturer’s instructions. RNA was then eluted in 50 μl RNAse-free water and its concentration assessed using a Nanodrop spectrophotometer and Qubit Fluorometer using the Qubit RNA Broad-Range (BR) Assay kit. Analysis of the integrity of the RNA was done using Agilent RNA 6000 Pico Kit and Eukaryotic Total RNA assay.

### Sequencing

Pacific Biosciences HiFi circular consensus DNA sequencing libraries were constructed according to the manufacturers’ instructions. Poly(A) RNA-Seq libraries were constructed using the NEB Ultra II RNA Library Prep kit. DNA and RNA sequencing was performed by the Scientific Operations core at the WSI on Pacific Biosciences SEQUEL II (HiFi) and Illumina HiSeq 4000 (RNA-Seq) instruments. Hi-C data were also generated from head tissue of ilApoLuen1 using the Arima v2 kit and sequenced on the Illumina NovaSeq 6000 instrument.

### Genome assembly, curation and evaluation

Assembly was carried out with Hifiasm ( Cheng *et al.*, 2021) and haplotypic duplication was identified and removed with purge_dups ( Guan *et al.*, 2020). The assembly was scaffolded with Hi-C data ( Rao *et al.*, 2014) using YaHS ( Zhou *et al.*, 2023). The assembly was checked for contamination as described previously ( Howe *et al.*, 2021). Manual curation was performed using HiGlass ( Kerpedjiev *et al.*, 2018) and Pretext ( Harry, 2022). The mitochondrial genome was assembled using MitoHiFi ( Uliano-Silva *et al.*, 2022), which performed annotation using MitoFinder ( Allio *et al.*, 2020). To evaluate the assembly, MerquryFK was used to estimate consensus quality (QV) scores and *k*-mer completeness ( Rhie *et al.*, 2020). The genome was analysed and BUSCO scores ( Manni *et al.*, 2021; Simão *et al.*, 2015) were calculated within the BlobToolKit environment ( Challis *et al.*, 2020). Table 3 contains a list of software tool versions and sources.

**Table 3.** Software tools: versions and sources.

Software tool	Version	Source
BlobToolKit	4.0.7	https://github.com/blobtoolkit/blobtoolkit
BUSCO	5.3.2	https://gitlab.com/ezlab/busco
Hifiasm	0.16.1-r375	https://github.com/chhylp123/hifiasm
HiGlass	1.11.6	https://github.com/higlass/higlass
Merqury	MerquryFK	https://github.com/thegenemyers/MERQURY.FK
MitoHiFi	2	https://github.com/marcelauliano/MitoHiFi
PretextView	0.2	https://github.com/wtsi-hpag/PretextView
purge_dups	1.2.3	https://github.com/dfguan/purge_dups
YaHS	yahs-1.1.91eebc2	https://github.com/c-zhou/yahs

### Genome annotation

The Ensembl gene annotation system ( Aken *et al.*, 2016) was used to generate annotation for the *Aporophyla lueneburgensis* assembly (GCA_932294355.1). Annotation was created primarily through alignment of transcriptomic data to the genome, with gap filling via protein-to-genome alignments of a select set of proteins from UniProt ( UniProt Consortium, 2019).

### Ethics and compliance issues

The materials that have contributed to this genome note have been supplied by a Darwin Tree of Life Partner. The submission of materials by a Darwin Tree of Life Partner is subject to the Darwin Tree of Life Project Sampling Code of Practice. By agreeing with and signing up to the Sampling Code of Practice, the Darwin Tree of Life Partner agrees they will meet the legal and ethical requirements and standards set out within this document in respect of all samples acquired for, and supplied to, the Darwin Tree of Life Project. All efforts are undertaken to minimise the suffering of animals used for sequencing. Each transfer of samples is further undertaken according to a Research Collaboration Agreement or Material Transfer Agreement entered into by the Darwin Tree of Life Partner, Genome Research Limited (operating as the Wellcome Sanger Institute), and in some circumstances other Darwin Tree of Life collaborators.

## Data Availability

European Nucleotide Archive: *Aporophyla lueneburgensis* (northern deep-brown dart). Accession number PRJEB50735; https://identifiers.org/ena.embl/PRJEB50735. ( Wellcome Sanger Institute, 2022) The genome sequence is released openly for reuse. The *Aporophyla lueneburgensis* genome sequencing initiative is part of the Darwin Tree of Life (DToL) project. All raw sequence data and the assembly have been deposited in INSDC databases. Raw data and assembly accession identifiers are reported in Table 1.

## Author information

Members of the University of Oxford and Wytham Woods Genome Acquisition Lab are listed here: https://doi.org/10.5281/zenodo.4789928.

Members of the Darwin Tree of Life Barcoding collective are listed here: https://doi.org/10.5281/zenodo.4893703.

Members of the Wellcome Sanger Institute Tree of Life programme are listed here: https://doi.org/10.5281/zenodo.4783585.

Members of Wellcome Sanger Institute Scientific Operations: DNA Pipelines collective are listed here: https://doi.org/10.5281/zenodo.4790455.

Members of the Tree of Life Core Informatics collective are listed here: https://doi.org/10.5281/zenodo.5013541.

Members of the Darwin Tree of Life Consortium are listed here: https://doi.org/10.5281/zenodo.4783558.
