# Supplementary material for: The genome sequence of the Northern Deep-brown Dart, Aporophyla lueneburgensis (Freyer, 1848)
Source: Wellcome Open Res. 2023 Mar 31;8:149. [Version 1] doi: 10.12688/wellcomeopenres.19296.1 (PMC10311123; doi:10.12688/wellcomeopenres.19296.1)
Supplement: Peer review history

# Peer review: Reviewer response for version 1

**DOI:** 10.21956/wellcomeopenres.21379.r61114

**Published:** 2023-06-29

**Authors:** Jesper Bechsgaard

**Affiliations:** 1 Department of Biology, Aarhus University, Aarhus, Denmark

The data note on the genome sequencing of the Northern Deep-brown Dart. The methods used are thorough and good, and the outcome is a high quality genome. The motivation to sequence the genome is not totally clear to me. The introduction is centered around previous mis-classification that seems to have been resolved. Even if not completely resolved, I feel that the authors should propose how whole-genome sequencing can help.

Are sufficient details of methods and materials provided to allow replication by others?

Yes

Is the rationale for creating the dataset(s) clearly described?

Partly

Are the datasets clearly presented in a useable and accessible format?

Yes

Are the protocols appropriate and is the work technically sound?

Yes

Reviewer Expertise:

Population genetics and genome evolution

I confirm that I have read this submission and believe that I have an appropriate level of expertise to confirm that it is of an acceptable scientific standard.

# Peer review: Reviewer response for version 1

**DOI:** 10.21956/wellcomeopenres.21379.r56053

**Published:** 2023-04-20

**Authors:** David Armisen

**Affiliations:** 1 Institut de Génomique Fonctionnelle de Lyon, Université Claude Bernard Lyon 1, Centre National de la Recherche Scientifique Unité Mixte de Recherche 5242, Ecole Normale Supérieure de Lyon, Universite de Lyon, Lyon, Auvergne-Rhône-Alpes, France

In this note the authors present the genome sequence of Northern Deep-brown Dart, *Aporophyla lueneburgensis*. The methods are sound and have been used numerous times by the the Tree of Life Consortium to produce high quality genomes as this one. The completeness and quality of the genome is very high and validated by various metrics such as k-mer completeness, BUSCO and percentage of assembly mapped to chromosomes. The note is straightforward and well-written and I only have very minor comments/suggestions. 

- I have particularly appreciated the discussion about *A. lueneburgensis *status as species through history. However, after such an introduction I was expecting more comparative analysis between both species that can highlight more the importance of whole genome sequencing. On the contrary, all controversy was already solved using mitochondrial DNA barcodes. Therefore I think that more importance could have been given to explain the interest to study colour pattern and specific food plant evolution in this species. 
- As a suggestion for future sequencing projects: The picture provided is of low quality and does not show the individual completely. Wings hide the body and the ventral side is not visible. I think that having more pictures of the sequenced individual (as supplementary material) could be helpful (see next point for an example). 
- The authors deduce that the sequenced individual is a Z0 female due to the half-coverage of the Z chromosome but no W chromosome found. While I agree with their interpretation, maybe in future genomes it would be worth observing the genitalia too. More pictures of the abdominal ventral section could have helped to verify this after sequencing.
- Another interesting information would be to have a karyotype from the sequenced species to confirm the 31 chromosomal-level scaffolds found by the authors.
- Methods section is extremely concise, without more details I assume that all the programs listed are used with default options. I think a phrase should be added to clarify that (hence the ‘Partly’ in sufficient details methods section). Otherwise, either a column could be added at Table 3 to include commands used, or a reference to an article with those details.

Are sufficient details of methods and materials provided to allow replication by others?

Partly

Is the rationale for creating the dataset(s) clearly described?

Yes

Are the datasets clearly presented in a useable and accessible format?

Yes

Are the protocols appropriate and is the work technically sound?

Yes

Reviewer Expertise:

Insect evolution, comparative genomics

I confirm that I have read this submission and believe that I have an appropriate level of expertise to confirm that it is of an acceptable scientific standard.
